# Acute graft versus host disease

**DOI:** 10.1186/1750-1172-2-35

**Published:** 2007-09-04

**Authors:** David A Jacobsohn, Georgia B Vogelsang

**Affiliations:** 1 Robert H Lurie Comprehensive Cancer Center and Division of Hematology/Oncology/Transplant, Children's Memorial Hospital, Chicago, IL, USA; 2 Sidney Kimmel Comprehensive Cancer Center, Johns Hopkins University School of Medicine, Baltimore, MD, USA

## Abstract

Acute graft-versus-host disease (GVHD) occurs after allogeneic hematopoietic stem cell transplant and is a reaction of donor immune cells against host tissues. Activated donor T cells damage host epithelial cells after an inflammatory cascade that begins with the preparative regimen. About 35%–50% of hematopoietic stem cell transplant (HSCT) recipients will develop acute GVHD. The exact risk is dependent on the stem cell source, age of the patient, conditioning, and GVHD prophylaxis used. Given the number of transplants performed, we can expect about 5500 patients/year to develop acute GVHD. Patients can have involvement of three organs: skin (rash/dermatitis), liver (hepatitis/jaundice), and gastrointestinal tract (abdominal pain/diarrhea). One or more organs may be involved. GVHD is a clinical diagnosis that may be supported with appropriate biopsies. The reason to pursue a tissue biopsy is to help differentiate from other diagnoses which may mimic GVHD, such as viral infection (hepatitis, colitis) or drug reaction (causing skin rash). Acute GVHD is staged and graded (grade 0-IV) by the number and extent of organ involvement. Patients with grade III/IV acute GVHD tend to have a poor outcome. Generally the patient is treated by optimizing their immunosuppression and adding methylprednisolone. About 50% of patients will have a solid response to methylprednisolone. If patients progress after 3 days or are not improved after 7 days, they will get salvage (second-line) immunosuppressive therapy for which there is currently no standard-of-care. Well-organized clinical trials are imperative to better define second-line therapies for this disease. Additional management issues are attention to wound infections in skin GVHD and fluid/nutrition management in gastrointestinal GVHD. About 50% of patients with acute GVHD will eventually have manifestations of chronic GVHD.

## Disease name and synonyms

Acute graft-versus-host disease or acute GVHD.

## Definition and diagnostic criteria

Acute graft versus host disease generally occurs after allogeneic hematopoietic stem cell transplant (HSCT). It is a reaction of donor immune cells against host tissues. The three main tissues that acute GVHD affects are the skin, liver, and gastrointestinal tract.

Clinically, the diagnosis is suspected when a recipient of HSCT develops any or all of the following signs or symptoms: dermatitis (skin rash), cutaneous blisters, crampy abdominal pain with or without diarrhea, persistent nausea and vomiting, hepatitis (with elevation of bilirubin and/or liver enzymes). Typically, these symptoms occur before day 100 after the HSCT, but may occur later. Symptoms most frequently start with donor engraftment, but may happen later. Acute GVHD is a clinical diagnosis but, as many the symptoms of acute GVHD are non-specific, histologic confirmation, especially if the symptoms are atypical or involve just the liver or gut, may be extremely useful.

Acute GVHD is staged by the number and extent of organ involvement. The current staging system was devised in 1994 (table 1) [1]. Recent data support the use of the grading system since it is able to subdivide patients into risk categories for complications and mortality. In this system, patients are divided into one of four grades (I-IV) depending on the degree, or stage, of involvement in three organs. The skin is staged with percent body surface involved, the liver is staged with degree of bilirubin elevation, and the gastrointestinal tract is staged with amount of diarrhea. Using the criteria (table 1), a single grade is assigned to each patient.

**Table 1:** Extent of organ involvement

**Stage**	**Skin**	**Liver (bilirubin)**	**Gut (stool output/day)**
**0**	No GVHD rash	< 2 mg/dl	< 500 ml/day or persistent nausea.
**1**	Maculopapular rash< 25% BSA	2–3 mg/dl	500–999 ml/day
**2**	Maculopapular rash 25 – 50% BSA	3.1–6 mg/dl	1000–1500 ml/day
**3**	Maculopapular rash > 50% BSA	6.1–15 mg/dl	Adult: >1500 ml/day
**4**	Generalized erythroderma plus bullous formation	>15 mg/dl	Severe abdominal pain with or without ileus
**Grade**			
I	Stage 1–2	None	None
II	Stage 3 or	Stage 1 or	Stage 1
III	-	Stage 2–3 or	Stage 2–4
IV	Stage 4 or	Stage 4	-

There is a similar grading system devised by the International Bone Marrow Transplant Registry (IBMTR). This sytem tries to diminish interobserver variability in GVHD grading. The system assigns one of four risk categories (A-D) to each patient with acute GVHD. It is still unknown whether one system is better at predicting outcome of patients with acute GVHD.

## Epidemiology

In 2003, 13,700 allogeneic HSCTs were reported to the Center for International Blood and Marrow Transplant Research (CIBMTR). The incidence of grade II-IV acute GVHD is roughly 35%–50%. The risk of GVHD increases with the use of unrelated donors, mismatched donor, older age of the donor, mutliparous female donor, older age of the recipient, graft type (cord blood has a lower rate than marrow or peripheral blood stem cells), and certain conditoning regimens. Given the current ratio of related *versus *unrelated donor transplants performed yearly (almost 2:1), we can expect about 5500 patients to develop grade II-IV acute GVHD per year.

## Clinical description

The clinical manifestations of acute GVHD represent the organs involved. The earliest and most common manifestation is skin GVHD. This is essentially a maculopapular rash that can begin anywhere in the body but often starts with palm and sole involvement. The patient may complain of pruritus or tenderness in affected areas. The onset of the rash normally correlates with engraftment of donor cells. The timing of engraftment depends on stem cell source (faster with peripheral blood stem cells) and intensity of preparative regimen. Patients receiving reduced intensity regimens, which do not result in marrow ablation, often have a later onset of GVHD. This is due both to the later engraftment and the damage from the preparative regimen producing cytokines that drive the immune responses resulting in clinical GVHD. As the rash progresses, it may become confluent. In severe cases, blisters may occur. The gastrointestinal manifestations include diarrhea which may become bloody, cramping, nausea, vomiting and failure to thrive [2]. Furthermore, jaundice from hyperbilirubinemia is the hallmark of liver GVHD [3], although a hepatitic variant of GVHD with a rise in liver enzymes like an acute viral hepatitis, has been recognized [4,5]. While acute GVHD is a clinical diagnosis, there are other conditions that can mimic or coexist with GVHD, such as drug toxicity (especially common in patients post transplant on multiple antimicrobial agents, immunosuppressive drugs, hyperalimentation, receiving methotrexate, *etc*.) and infection. For example, the symptoms of cytomegalovirus (CMV) colitis (diarrhea, abdominal pain) may be very similar to those of acute GVHD. Therefore, it is recommended to obtain a biopsy to confirm clinical suspicion whenever possible. Finally, while the definition of acute GVHD is well-accepted as involving three organs, certain post-transplant complications may be related to or actually represent GVHD. For example, a few patients develop non-infectious pulmonary infiltrates upon engraftment and the outcome is generally poor. Animal models suggest that the immunologic mechanisms contributing to lung inflammation after HSCT may be similar to those responsible for GVHD [6].

## Etiology

**Figure 1:**
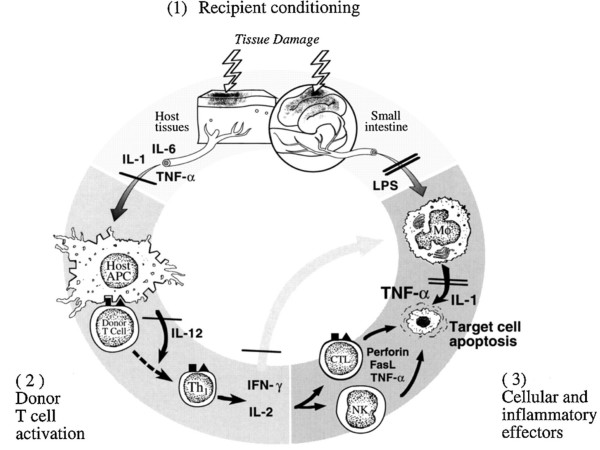
The three phases of acute GVHD, as described by Ferrara and colleagues. (from: Hill GR, Ferrara JLM. The primacy of the gastrointestinal tract as a target organ of acute graft-versus-host disease: rationale for the use of cytokine shields in allogeneic bone marrow transplantation. Blood 2000;95:2754–2759. ***Copyright American Society of Hematology, used by permission)***.

The pathophysiology of acute GVHD has been described by Ferrara and colleagues as a three-phase phenomenon [7,8]. Please refer to figure 1. The first involves damage to host tissues by inflammation from the preparative chemo- and/or radio-therapy regimen. In the second phase, both recipient and donor antigen-presenting cells (APCs) as well as inflammatory cytokines triggering the activation of donor-derived T cells, which expand and differentiate into effector cells [9]. In this activation phase, minor histocompatibility antigens play a central role, particularly in the setting of matched sibling transplantations.

T-cell activation pathways result in the transcription of genes for cytokines, such as IL-2 and interferon. T cells that produce IL-2 and interferon are considered to be of the Th1 phenotype, compared to T cells that produce predominantly IL-4, IL-5, IL-10, and IL-13, which define the Th2 phenotype, that are felt to modulate GVHD [10].

In the third phase, the effector phase, activated donor T cells mediate cytotoxicity against target host cells through Fas-Fas ligand interactions, perforin-granzyme B, and the additional production of cytokines, such as TNF-**α **TNF-**α **is produced mainly by monocytes and macrophages, and secondarily by T lymphocytes and natural killer cells. TNF-**α **has been implicated in the pathophysiology of GVHD at several steps in the process, including induction of apoptosis in target tissues through the TNF-**α **receptor; activation of macrophages, neutrophils, eosinophils, B cells, and T cells; stimulating production of additional inflammatory cytokines (IL-1, IL-6, IL-10, IL-12, and TNF-**α **itself); increased expression of HLA; and the facilitation of T-lymphocyte lysis. High levels of TNF-**α **also have been associated with an increased incidence of GVHD in bone marrow transplantation (BMT) recipients. This allogeneic interaction in the setting of cytokine dysregulation leads to the tissue damage characteristic of acute GVHD [9,11-15].

## Risk factors

### Genetic basis of acute GVHD

Major histocompatibility antigens (or Human leukocyte antigens – HLA) are located on the short arm of chromosome 6 at the p21 position in humans [16,17]. The major histocompatibility complex (MHC) is a closely linked, highly polymorphic multi-gene and multi-allelic complex playing the critical role in both cell-mediated and humoral immune responses. HLA class I antigens (HLA-A, HLA-B, and HLA-C) have a wide distribution and are found on all nucleated cells. HLA class II antigens (DR, DQ, and DP) are generally found on the cells involved in the immune response. CD4 T cells recognize foreign antigens by the presentation of class II HLA molecules. Interestingly, class II HLA products are particularly induced on the skin and intestinal tract epithelial tissues, they may promote specific targeting during acute GVHD. Matching BMT recipients with sibling donors sharing identical HLA antigens improves engraftment and decreased GVHD compared to mismatched siblings. However, acute GVHD is seen in matched sibling pairs-demonstrating factors contribute to the immune reaction. Minor histocompatibility antigens (miH) are peptides derived from intracellular proteins presented by MHC molecules to donor T cells. Perhaps the best known of these are the minor antigens from products from the Y chromosome. Alloreactivity in the setting of matched siblings then involves the recognition of different nonself peptides bound in the T cell receptor and carried by the recipient MHC. Following the presentation of miH (foreign peptide) by MHC to donor T cell, *i.e*., CD4 in the context of MHC class II and CD8 in class I, the presence of nonself peptide bound to the MHC molecules trigger the T cell and induction of GVHD occurs. Current research is focused on identification of additional non-MHC antigens that trigger alloreactivity.

With the ability to molecularly type the extensive HLA region, it became evident that much more of the HLA region had to be considered for optimal unrelated donor selection. This is an area of explosive development, with almost 200 new alleles identified each year. A detailed review of the intricacies of the HLA system and selection of donors is beyond the scope of this paper. There are several recent excellent reviews [16,17]. However, a few guiding principles can be stated. The most important is that it is critical to have an experienced transplant immunogeneticist review all genetic data. Second, regions that were not appreciated to have a role in GVHD (plus engraftment and graft-versus-leukemia, GVL) in the matched sibling transplants are clearly important in the unrelated/mismatched family donor. For example, the HLA-C and HLA-DQ regions are routinely typed when looking for an alternative donor (the so called 10 antigen match). More recently, HLA-DP and HLA linked NK cell typing has been shown to play a role in the GVHD/GVL equations. Interestingly, data collected on unrelated donor transplants indicates, there is evidence that certain allelic mismatches are „permissible“-that is, although the alleles are molecularly different, the actual immune consequences are modest to undetectable. It is also increasingly apparent that the balance of GVH/GVL is shifted by the intensity of the preparative regimen-so that the best match for an unrelated donor transplant receiving a full preparative regimen may be different from that in a non-myeloablative setting. The complexities of the HLA system demonstrate why it is critical to have the input of an experienced transplant immunogeneticist.

### Other risk factors

The risk factors for acute GVHD are well defined. As discussed above, the most important factor is HLA disparity. Among siblings, patients receiving matched grafts have lower rates of GVHD than those receiving one, two or three antigen mismatch grafts. For unrelated donor transplants, the greater the degree of HLA-mismatch, the higher the likelihood is of developing acute GVHD and the worse the overall outcome [18-20]. Recent data from the National Marrow Donor Program suggests that matching at the allele level (high-resolution) as opposed to only at the antigen (low-resolution) level provides advantage in reducing the likelihood of GVHD. The incidence of serious (grade III/IV) acute GVHD is about 30% with a fully matched (8/8) unrelated donor but is 40% with 1 or 2 allelic mismatches at class I [21,22]. This compares to about a 20% incidence of serious acute GVHD for recipients of HLA-identical sibling transplants [23].

As for the source of the graft, unrelated cord blood has become an important alternative stem cell source and has some unique properties. The immunologic naiveté of these stem cells allows for greater degrees of mismatch; recipients of mismatched (4/6 or 5/6 HLA group match) unrelated cord blood appear to have similar incidence of acute GVHD and similar overall outcome as compared to matched-sibling transplants [24]. There is increasing use of peripheral blood stem cell (PBSC) as a way of collecting cells from related or unrelated donors. No randomized study has been completed to determine if PBSC transplants change GVHD incidence or eventual outcome. However, there is a suggestion from a meta-analysis that acute GVHD is slightly increased (RR 1.16) and chronic GVHD is increased (RR 1.53) when comparing PBSC to bone marrow transplants [25]. Higher doses of CD34+ cells in the PBSC product correlate with more GVHD [26]. It is still debated whether the increase in GVHD will translate to improved outcome due to higher graft-versus-leukemia effect or worse outcome due to higher transplant-related-mortality. Some groups have used T-cell depletion which decreases the risk of acute GVHD [27-29]. However, this approach has led to an increased incidence of graft failure and slower immune reconstitution. Clinically this has translated to more infection and relapse.

Other factors can also increase the likelihood of acute GVHD. Older age of both recipient and donor increase the probability of GVHD [30]. Sex mismatch, specifically a multiparous female donor into a male patient, increases the likelihood of GVHD [31]. Furthermore, the intensity of the preparative regimen does appear to correlate with more acute GVHD. This effect may occur to more tissue damage from the preparative regimen predisposing these tissues to more inflammation from the alloreactive cells. Higher doses of radiation do give rise to more GVHD [32], and the more recent use of nonmyeloablative preparative regimens has led to lower incidence of acute GVHD in some studies [33-35].

## Diagnostic methods

Acute GVHD is usually suspected based on the clinical presentation. A biopsy can be used to confirm the diagnosis and should be used when there are competing diagnoses, such as infection and drug reaction, in the differential. Skin biopsies can show dyskeratotic keratinocytes, lymphocyte exocytosis, basal cell necrosis, depletion of Langerhans cells, and satellite lymphocytes next to the dyskeratotic keratinocytes. All findings are not necessarily present in every skin biopsy and histology is not always pathognomonic [36]. Typically, endoscopy of the gastrointestinal tract reveals edema, mucosal sloughing and possibly bleeding. Most typically these would be found in the cecum, ileum and colon but also may involve the upper intestinal tract. Histopathology shows crypt-cell necrosis and dropout with crypt absecess [37]. Pathology of liver GVHD can show early cytotoxic lymphocyte attack on bile ducts to bizzare, irregular bile ducts, depending on the timing of the biopsy *vis a vis *the duration of liver GVHD prior to the biopsy. Epithelial cells can be flattened with some missing nuclei. Other nuclei are often enlarged, irregular, and hyperchromatic. Bile duct apoptosis and endothelialitis can also be seen [38].

## Management

Currently, most centers use a combination of an immunophilin (cyclosporine or tacrolimus) with short course methotrexate. Although other regimens are being explored, this particular regimen has been shown repeatedly to result in a reasonable balence of GVHD and graft versus-tumor in matched sibling transplants after ablative chemotherapy [39,40]. For higher risk groups or groups receiving non-conventional grafts (such as mismatched donors, older patients, reduced intensity regimens, *etc*.), the best prophylaxis is less clearly established [41]. Mycophenolate mofetil (MMF) is often used in reduced intensity transplantation for its effect in GVHD prophylaxis as well as promoting engraftment [42]. For patients reciving mismatched grafts, more intensive immunosuppression is usually needed. Methods of *ex-vivo *T-cell depletion [43] as well as pharmacologic *in-vivo *T cell depletion (antithymocyte globulin, ATG [44], alemtuzumab [45]) have been used to attempt to reduce the incidence of acute GVHD. In general, these methods reduce acute GVHD but increase the incidence of infection (due to delayed reconstitution of the immune system) and the incidence of relapse (due to a decreased graft-versus-leukemia effect).

Once GVHD occurs, all phases of GVHD induction are active. Successful treatment will ultimately need to work on all phases, if the process is to be stopped. Most centers treat grade II-IV acute GVHD by continuing prophylactic immunosuppression and adding methylprednisolone at 2 or 2.5 mg/kg/day. However, starting doses range from 1 to >20 mg/kg/day [46]. Steroids are tapered after control of GVHD. A rapid steroid taper (86 days) is just as effective as a slow taper (147 days) in terms of preventing flares of GVHD or chronic GVHD [47]. A few studies have reported outcomes with high dose methylprednisolone (20 to 50 mg/kg/day). Patients that responded to these doses generally flared after dose reduction, and there were a number of deaths secondary to opportunistic infections [48,49].

There is one randomized trial comparing high and low dose methylprednisolone for the treatment of acute GVHD [50]. Patients receiving 2 mg/kg/day and 10 mg/kg/day had the same rate of response (70%) and the same 3-year actuarial survival (62%). Higher morbidity was observed with the higher dose. Therefore, there is no compelling argument to use the super high dose of steroids. Initial response to steroids, either low-dose or high-dose, is very predictive of future severity of GVHD and other transplant complications. In general, about 40–50% of patients have an overall response to corticosteroids [51].

Patients not responding to corticosteroids are treated with salvage therapy. There is no specific approach that is considered standard of care. Anti-thymocyte globulin has been used, and produces objective responses. However, the long-term survival of patients treated with ATG is low (median survival 4.1 months) given the severe immunosuppression and high incidence of infection [52]. There are a number of other approaches under investigation. Some of these include extracorporeal photopheresis [53], pentostatin [54,55], sirolimus [56], infliximab[57], and mesenchymal stem cells [58]. In general, a number of these agents produce responses; however, infectious mortality remains high. Systematic investigation of dosing and timing of various salvage agents is clearly necessary in well-designed, prospective clinical trials.

While the goal is not to review all potential salvage therapies, a few types will be mentioned (Deeg recently published an excellent review on therapies for steroid-refractory GVHD [59]). For example, of twenty-two heavily-treated patients with steroid-refractory acute GVHD treated with pentostatin (an irreversible inhibitor of adenosine-deaminase) on a phase I trial, seventeen had an objective response. However, five patients that responded died from late infections, either viral or fungal. The survival at one year was 25% [55]. Possibly, employing a salvage approach such as this one earlier in the process of acute GVHD may produce an improved outcome.

Monoclonal antibodies such as daclizumab and infliximab have been used to treat GVHD. MD Anderson Cancer Center recently reviewed their experience using infliximab (anti-TNF-**α **antibody) in 21 patients with steroid-refractory acute GVHD. This was the only drug added to these patients. Sixty-seven percent responded, and there was significant activity in gastrointestinal GVHD. There was a high rate of infections, particularly fungal and viral. One-year survival was 38% [60]. Another study compared patients with steroid-refractory acute GVHD that received infliximab *versus *those that did not, and found a higher incidence of non-*Candida *invasive fungal infections in those receiving infliximab [61]. Thus, while this and other drugs may offer responses to patients with GVHD, it is always important to remember that by far the greatest risk will be increased infectious complications.

Additional issues for patients with GVHD are appropriate management of symptoms. For example, patients with severe gastrointestinal GVHD and diarrhea need careful attention to fluid status, electrolyte management and protein-losing enteropathy. Patients with skin GVHD need to be thoroughly examined for the presence of any open sores or bullae, which may become infected. Since infectious complications are so prevalent in these patients, frequent monitoring of CMV PCR or antigenemia and appropriate therapy is important [62]. Published CDC (Centers for Disease Control and Prevention) guidelines for prevention of infection (pneumcystis jirovecii pneumonia, bacterial, fungal, viral) should be closely followed [63].

## Prognosis

MacMillan *et al*. from the University of Minnesota recently published the response of 443 patients with acute GVHD, treated uniformly at their institution. Of the 443 patients (all treated with prednisone), durable responses were obtained in 245 (55%). There was a tendency to lower response if patients begun with a higher grade. Recipients of HLA-mismatched unrelated donor transplants were less likely to respond. Fifty-three percent of patients were alive at 1 year after initiation of steroid therapy, and 42% of patients had developed chronic GVHD. Deaths were mostly commonly attributed to ongoing GVHD and/or infection [64]. Cahn and colleagues recently reported on a multicenter study comparing the IBMTR and the Glucksberg scales. Both scales performed similarly. In general, patients with grade C (IBMTR) or grade III (Glucksberg) acute GVHD have about a 30% probability of long-term survival. Those with grade D/grade IV acute GVHD, have under 5% long-term survival. Patients without GVHD or with grade A-B/grade I-II acute GVHD have above 80% probability of long-term survival [65].

It is worth noting chronic GVHD as over half of the patients with acute GVHD later develop chronic GVHD. Chronic GVHD is a distinct clinical syndrome, although there may be an overlap period, where the pateint has symptoms of both acute and chornic GVHD. Chronic GVHD resembles many spontaneously occuring autoimmune disorders, like scleroderma. Organs affected are most typically skin (lichenoid and sclerotic rashes), mouth, joints, liver, eyes, gastrointestinal tract, and occasionally lungs [66]. While chronic GVHD can worsen survival due to more transplant-related mortality (infection from immunosuppression), chronic GVHD can also have a GVL effect. The basic pathophysiology of chronic GVHD is at this point not well defined. Therapy relies on many of the same medications used to treat acute GVHD, but patients require prolonged treatment, extending over months to years [67]. Despite the fascinating clinical manifestations of chronic GVHD, the most important aspect of chronic GVHD is the significant immune dysfunction associated with the disease itself and its treatment. Infection accounts for the majority of deaths in chronic GVHD patients.

## Unresolved questions

Clearly, better immunosuppressive therapies are needed for patients with severe GVHD. In addition, as immunosuppression is increased in these patients, the risk of infection needs to be carefully weighed. Agents for prevention of acute GVHD and for treatment of steroid-refractory acute GVHD need further investigation. To be able to truly evaluate the potential clinical benefit of an agent, it needs to be studied in prospective clinical trials. Furthermore, it is imperative to continue investigations on methods of cellular manipulation (selective T cell depletion, alloreactive NK cell infusions) that may reduce GVHD while preserving the graft-versus-leukemia effect. Since GVHD is clearly the major barrier to successful HSCT, efforts are needed in this specific area. If GVHD outcomes begin to improve, we may see an increase in number of patients being referred for HSCT.
